# Navigating the Tumor Microenvironment in Colorectal Liver Metastasis: Barriers to Therapy and Emerging Opportunities

**DOI:** 10.32604/or.2026.076013

**Published:** 2026-04-22

**Authors:** Pengtao Hu, Junjie Sun, Jian Lu, Chunlei Ge, Hanzhi Sun, Chengyu Lv

**Affiliations:** 1 Department of General Surgery, Nanjing First Hospital, Nanjing Medical University, Nanjing, China; 2 International Oncology Institute, the First Affiliated Hospital of Zhejiang Chinese Medical University, Oncology Department of the First Affiliated Hospital of Zhejiang Chinese Medical University, Hangzhou, China; 3 Department of General Surgery, Gaochun Hospital of Traditional Chinese Medicine, Nanjing, China

**Keywords:** Colorectal cancer, liver metastasis, tumor microenvironment, pre-metastatic niche, immuno-suppression, targeted therapy

## Abstract

Liver metastases from colorectal cancer (CRC) are a primary cause of poor patient prognosis, closely linked to the liver’s unique tumor microenvironment (TME). Compared to primary tumors, research on the TME of liver metastases remains insufficient. This review systematically summarizes recent advances in TME research concerning colorectal liver metastases (CRLM), emphasizing its organ-specific characteristics, pivotal role in tumor progression, and influence on treatment response. We delve into the intricate cellular components of the TME—including tumor-associated macrophages, cancer-associated fibroblasts, and myeloid-derived suppressor cells—and non-cellular constituents such as the extracellular matrix and soluble factors. Furthermore, we explore the multifaceted mechanisms which the TME drives CRLM progression through establishing pre-metastatic niches, facilitating cancer cell colonization, mediating immune evasion, and inducing drug resistance. Additionally, we evaluate therapeutic strategies targeting the TME, including opportunities and challenges in remodeling cellular components, modulating the extracellular matrix, and developing combination therapies. Ultimately, this review aims to provide theoretical foundations and novel insights for developing more effective anti-metastatic therapies, with the goal of improving the prognosis for CRLM patients.

## 1 Introduction

**Figure 1:**
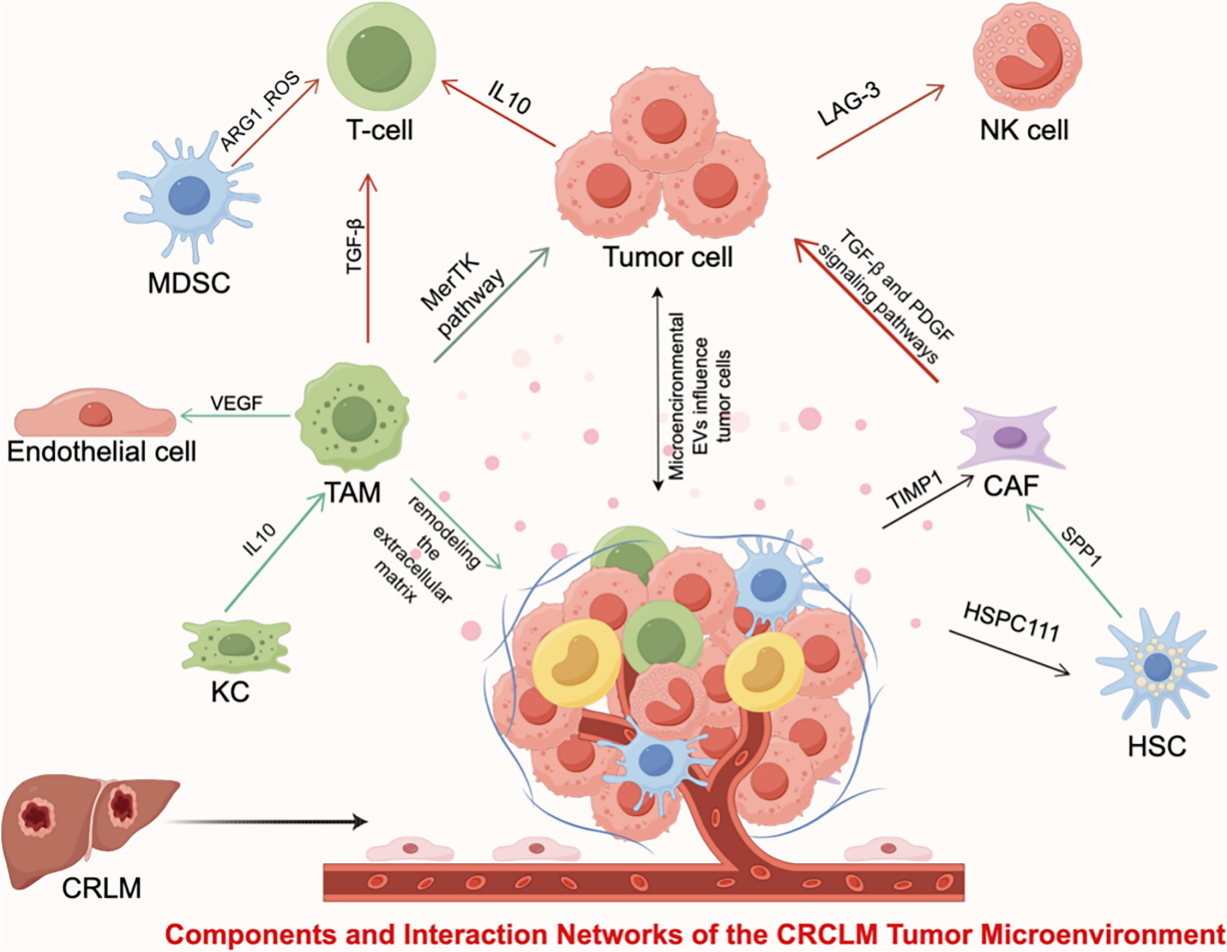
Schematic representation of tumor microenvironment components in colorectal cancer liver metastases. The core composition of TME includes tumor-associated macrophages (TAMs), cancer-associated fibroblasts (CAFs), Myeloid-derived suppressor cells (MDSCs), hepatic stellate cells (HSCs), and kupffer cells (KCs). This schematic illustrates the cellular composition and intercellular communication network within the TME of CRLM. Tumor cells are positioned at the center and function as the major signaling hub, dynamically interacting with immune cells, stromal cells, and vascular components through cytokines, growth factors, metabolic mediators, and extracellular vesicles (EVs), thereby shaping an immunosuppressive and pro-metastatic niche in the liver. Abbreviations: ARG1, Arginase 1; CRLM, Colorectal liver metastasis; HSPC111, Hematopoietic stem cell precursor 111; IL-10, Interleukin-10; LAG-3, Lymphocyte-activation gene 3; NK cell, Natural killer cell; PDGF, Platelet-derived growth factor; ROS, Reactive oxygen species; SPP1, Secreted phosphoprotein 1; T-cell, T lymphocyte; TGF-β, Transforming growth factor-beta; TIMP1, Tissue inhibitor of metalloproteinases 1; VEGF, Vascular endothelial growth factor.

Colorectal cancer (CRC) is the third most prevalent malignancy worldwide and the second leading cause of cancer-related mortality [1]. Despite the gradual improvement in the prognosis of patients diagnosed with CRC in high-income countries over the past several decades, metastatic CRC (mCRC) remains associated with a dismal survival rate of less than 15% with the liver being the most common site of metastasis [2,3]. Although some patients with colorectal cancer liver metastases (CRLM) may benefit from therapies that target the epidermal growth factor receptor (EGFR) and vascular endothelial growth factor receptor (VEGFR)-targeted signaling pathways, surgery is the only treatment that has the potential to cure colorectal liver metastases [4]. In recent studies, immune checkpoint inhibitors (ICIs) have demonstrated substantial clinical benefits in CRCs with high microsatellite instability or mismatch repair deficiencies [5,6]. This progress shows the great potential of therapies that target the tumor microenvironment (TME). This field has begun to understand the critical factors that determine mCRC (Fig. 1).

Recent findings about the TME have revealed that cancer metastasis to specific sites can be attributed to a microenvironment that functions as a trap for tumor cells [7]. The TME is a complex ecosystem composed of non-malignant cells (such as Cancer-associated fibroblasts (CAFs), immune cells, endothelial cells, etc.), extracellular matrix (ECM), and their secreted molecules, playing a decisive role in tumorigenesis, metastasis, and treatment response [8,9]. As tumors grow, they actively rewire their surroundings. The TME breaks down the healthy tissue’s architecture—both biochemically by creating hypoxia and mechanically by increasing stiffness—which in turn drives malignant transformation [9,10]. TME heterogeneity (e.g., immunosuppressive cell infiltration) and dynamic mechanical forces also induce immunotherapy resistance [11]. The TME has emerged as a critical therapeutic target [8,12], with microfluidic tumor chips and artificial tumor models (tumoroids) employed to mimic the TME for drug screening and mechanism studies [13,14]. Recent studies have further elucidated the complexity of the TME in CRLM, highlighting unique cellular crosstalk and potential therapeutic vulnerabilities [15–18]. Strategies targeting CAFs or ECM stiffening offer novel directions for cancer therapy [8,19].

Although primary CRCs that can spread to other parts of the body have different immune and stromal features, we still do not fully understand how the TME contributes to metastasis. In recent years, an increasing number of descriptive studies have employed single-cell transcriptomics to compare the TME composition of liver metastases with that of primary colorectal cancer [20–22]. However, we still need to understand the role of TME in CRLM better. This review systematically maps the complex network of the TME in CRLM, explores its crucial role across metastatic stages, and envisions future personalized treatment methods based on TME modulation.

Given these complexities, and in alignment with ICMJE recommendations, this review aims to provide a holistic characterization of the organ-specific TME in CRLM. Specifically, we seek to dissect the intricate cellular and non-cellular architecture unique to the hepatic niche, elucidate how these components collaboratively drive immune evasion and therapeutic resistance, and critically appraise emerging TME-targeted interventions. Ultimately, this work is intended to bridge the gap between basic TME biology and clinical practice, offering a robust theoretical foundation to bypass existing hurdles and improve the prognosis for patients with CRLM.

## 2 The Intricate Composition of the TME in CRLM

### 2.1 Histopathological Growth Patterns of CRLM: Implications for the TME

**Figure 2:**
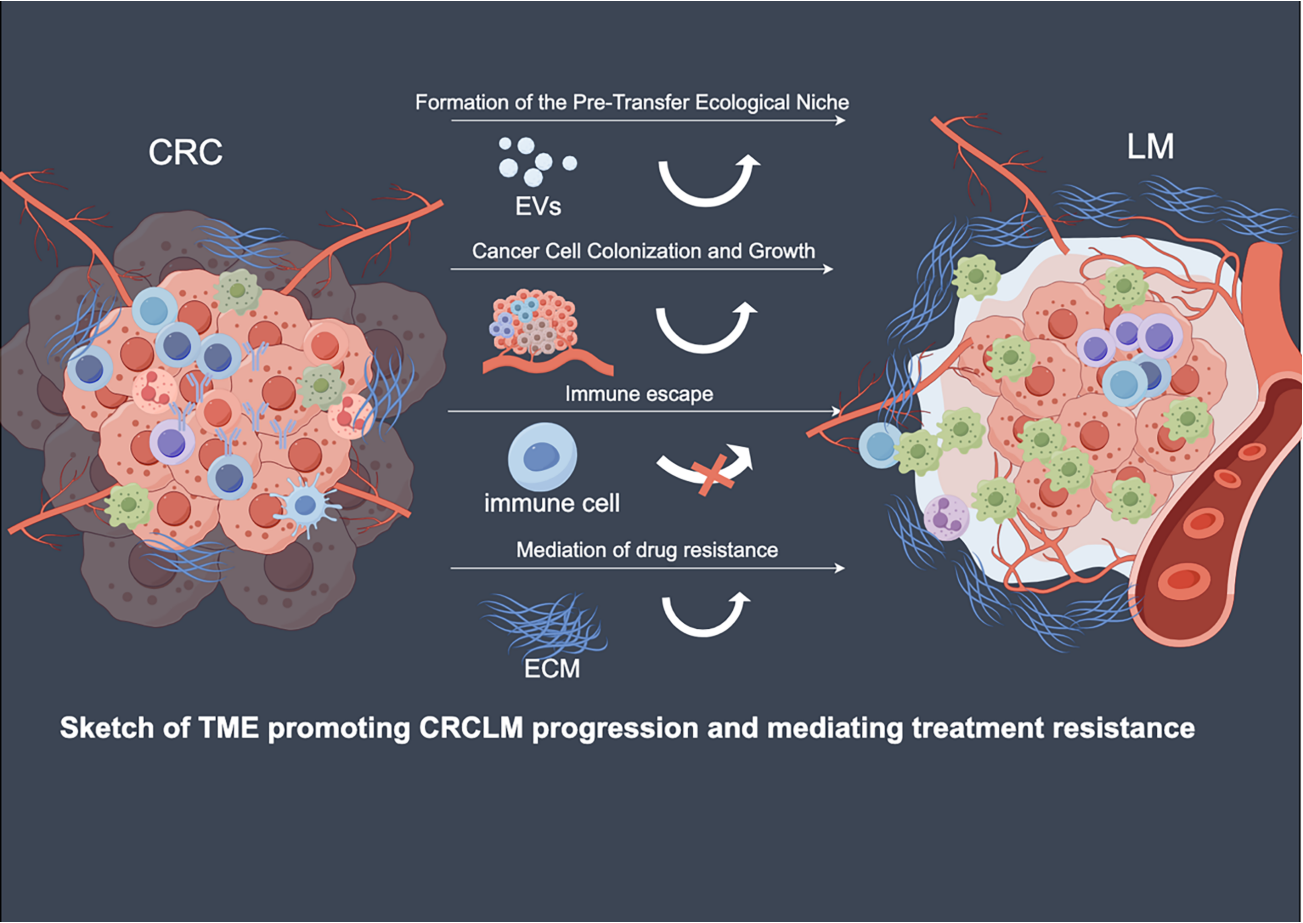
Mechanism diagram of TME promoting the progression of CRLM and mediating treatment resistance. Here, we present the most significant mechanisms in the formation of CRLM. They jointly participate in the liver metastasis of colorectal cancer through mechanisms such as altering the pre-metastatic niche, enhancing the colonization of cancer cells, and inducing immunosuppression. CRC, Colorectal cancer; CRCLM, Colorectal cancer liver metastasis; ECM, Extracellular matrix; EVs, Extracellular vesicles; LM, Liver metastasis; TME, Tumor microenvironment.

The interaction between metastatic cancer cells and the liver parenchyma gives rise to distinct histopathological growth patterns (HGPs), which are crucial for understanding TME heterogeneity and clinical outcomes [23]. These patterns primarily include the replacement HGP, where cancer cells co-opt the pre-existing liver sinusoidal structure and replace hepatocytes, and the desmoplastic HGP, characterized by a fibrous capsule separating the tumor from the liver parenchyma with new vessel formation at the interface (Fig. 2) [24].

The choice of HGP is not random but is dictated by the dynamic crosstalk within the TME. The replacement HGP is associated with prominent angiogenic signaling and a more immunosuppressive TME, often featuring higher infiltration of M2-polarized TAMs and poorer response to anti-angiogenic therapies like bevacizumab [25]. In contrast, the desmoplastic HGP typically exhibits a stronger lymphocyte infiltrate at the invasive margin, which correlates with a better prognosis and may respond more favorably to immunotherapy [26].

Therefore, recognizing HGPs is essential for a nuanced understanding of the CRLM TME. It provides a pathological framework that links specific cellular and molecular features of the TME to patient survival and therapy response, underscoring the need for personalized treatment strategies based on not only molecular but also pathological TME subtypes.

### 2.2 Cellular Components

#### 2.2.1 Tumor-Associated Macrophages (TAMs)

**The origin and phenotypic plasticity of TAM (M1/M2)**

The origin of macrophages can be traced back to either local tissue-resident macrophages, which possess a self-renewal ability, or blood monocytes, which subsequently transform into macrophages [27]. Macrophages within the TME exhibit notable plasticity and heterogeneity. There are two types of macrophages: M1-type and M2-type. M1-type macrophages have pro-inflammatory, immune-activating, and anti-tumor properties. M2-type macrophages express immunosuppressive molecules, indicating poor prognosis in CRC and a high concentration in liver metastases [28,29].

**Immunosuppressive roles and pro-angiogenic effects**

M2-type TAMs promote immune suppression through multiple mechanisms: directly inhibiting CD8^+^ cytotoxic T lymphocytes (CD8^+^ T cells) cell activity and reducing their infiltration, promoting Treg expansion by secreting **i**nterleukin-10 (IL-10) and transforming growth factor-beta (TGF-β), and exacerbating immunosuppression via MerTK (c-mer proto-oncogene tyrosine kinase)-mediated phagocytosis [30,31]. Concurrently, TAMs help new blood vessels to form by releasing factors like vascular endothelial growth factor (VEGF) and fibroblast growth factor 2 (FGF2). These cells begin to interact directly with vascular endothelial cells early in the metastatic process [11,32,33], and also participate in remodeling the extracellular matrix by secreting enzymes such as matrix metalloproteinases, which is essentially a pre-metastatic preparation [34]. This advanced prognostic role makes TAMs a highly promising biomarker, as they can be tracked in future clinical trials for their density and phenotype, thereby helping predict a patient’s response to chemotherapy or immunotherapy.

**Mediation of drug resistance**

TAMs are closely related to chemotherapy resistance. In general, M2-polarized TAMs create an immunosuppressive state in cancer cells by releasing protective factors such as IL-10 and TGF-β [32,35,36]. Metabolic imbalances in the tumor microenvironment often drive this immunosuppressive M2 state. Usually, the accumulation of lactic acid leads to a localized accumulation in the tumor, which further weakens the antitumor response [37,38]. However, this mechanism is reversible. Some studies have demonstrated improved drug resistance through intervention in M2 subpopulations [34,39].

#### 2.2.2 Cancer-Associated Fibroblasts (CAFs)

**The origin and phenotypic diversity of CAFs**

CAFs primarily originate from tissue-resident fibroblasts (also known as quiescent fibroblasts) [40], mesenchymal stem cells (MSCs) [41], and adipocytes [42]. Tissue-resident fibroblasts are a primary source of CAFs. Resident fibroblasts in different tumor tissues are sequentially recruited and activated by continuous stimuli that stimulate various modulators, such as TGF-β and platelet-derived growth factor (PDGF) signaling pathways, thereby creating a specific environment conducive to tumor growth [43]. Interestingly, the liver’s unique immune environment may allow hepatic stellate cells (HSCs) to serve as an additional source of CAFs [44]. CAFs are not static players; they interact with other components of the tumor microenvironment and dynamically evolve during tumor progression in response to signals from the surrounding microenvironment [45].

**The Role of CAFs in cytokine secretion, immunoregulation, and drug delivery impairment**

CAFs have crucial roles in managing immune cells in tumors. This adaptability enables them to perform critical functions. CAFs directly influence innate immune cells, as well as adaptive immune cells [46]. Also, they can promote an immunosuppressive environment by driving immune checkpoint molecule expression and remodeling the extracellular matrix, roles that indirectly shape immune cell recruitment and function [47]. CAFs can encourage immune cells to take part in the development and growth of cancer by releasing molecules like cytokines, chemokines, TGF-β, and collagen [47,48]. CAFs can make it hard for tumor-killing cells and therapeutic agents to infiltrate and exit the tumor tissue [49].

#### 2.2.3 Myeloid-Derived Suppressor Cells (MDSCs)

**Classification of MDSC**

MDSCs are a group of immature myeloid cells that include different types of cells. There are two main types of MDSCs: granulocytic or polymorphonuclear (PMN-MDSC) and monocytic (M-MDSC) [50]. PMN-MDSCs significantly increase in SMAD family member 4 (SMAD4)-deficient liver metastases via the C-C motif chemokine ligand 15 (CCL15)/C-C motif chemokine receptor 1 (CCR1) and CCL9/CCR1 axes, with single-cell sequencing revealing their strongest immunosuppressive activity [51,52]; Monocytic myeloid-derived suppressor cells (M-MDSCs) are closely associated with immune checkpoint inhibitor resistance [53].

**Immunosuppression mechanism**

MDSCs directly damage CD8^+^ T cells, inducing T cell death [54,55], while promoting the expansion of regulatory T cells (Tregs) [54,56]. MDSC also blocks T cell activation by secreting metabolites such as arginase 1 (ARG1) and reactive oxygen species (ROS) [57]. In addition to targeting T cells, MDSCs also enhance tumor invasiveness by participating in epithelial-mesenchymal transition (EMT) and chemotherapy resistance (often via the interleukin-23 (IL-23)/signal transducer and activator of transcription 3 (STAT3) pathway) [58]. Its immunosuppressive effects are exerted through synergistic interactions with TAMs, creating a constitutive microenvironment locally in tumor suppression [51,59]. This inhibitory function is also further enhanced by the hypoxia-inducible factor-1 alpha (HIF-1 α) signaling pathway under hypoxic conditions, a common feature of tumors [60].

**Role in CRLM progress**

MDSCs infiltrate liver metastases at an early stage, and they promote tumor colonization and immune escape by inhibiting natural killer cell and T-cell function [61,62], especially in SMAD4-deficient models, where CCR1^+^ Granulocytic myeloid-derived suppressor cells (G-MDSCs) infiltration is significantly associated with T-cell dysfunction [52]. MDSC accumulation has also been found to be strongly associated with poor patient prognosis [63,64].

**Immune therapy resistance**

MDSCs also significantly promote resistance to immunotherapy: elevated peripheral blood MDSC levels before anti-programmed cell death protein 1 (anti-PD-1) treatment predict poor response [65]; they attenuate PD-1/programmed cell death ligand 1 (PD-L1) blockade by recruiting Tregs and depleting CD8^+^ T cells, and synergistically maintain the “cold tumor” phenotype through interactions with tumor exosomes [66].

#### 2.2.4 Hepatic Stellate Cells (HSCs) and Kupffer Cells (KCs)

**The liver’s “native” cells**

In CRLM, the liver-resident macrophage population, Kupffer cells (KCs), exhibit dual roles: on one hand, cytokines secreted by CRC cells can induce KCs to polarize toward an M2 phenotype, promoting metastasis by secreting pro-inflammatory factors such as TGF-β [67,68]. On the other hand, under normal conditions, KCs resist metastasis by phagocytosing circulating tumor cells. Still, tumor-derived small extracellular vesicles (EVs) can suppress their antitumor activity by interfering with the apoptotic protease activating factor 1 (APAF1)-dependent DNA damage response [69,70].

In CRLM, HSCs are activated and transformed into CAFs, which promote fibrosis in the local tumor microenvironment through the secretion of collagen (e.g., Col1α1) and Timp1. This change provides structural support for metastasis [71,72].

**The unique role of CRLM in seeding and growth**

Activated KCs further stimulate HSCs by releasing TGF-β1. For instance, Lipopolysaccharide-stimulated KCs upregulate CCL2 and Timp1 expression in HSCs while inhibiting MMP1 [72]. In turn, type I collagen secreted by HSCs activates the TGF-β1 signaling pathway via the DDR1 receptor. These changes in the hepatic pro-local immune microenvironment lead to the survival of tumor cells and maintenance of stem cell properties [73].

Regarding intercellular communication and microenvironment regulation, tumor-derived EVs simultaneously target KCs and HSCs: DNA-carrying EVs activate the DNA damage response in KCs. At the same time, lipid metabolites (e.g., PA/OA) promote fibrosis progression via KC-HSC co-culture models [74]. Proinflammatory factors released by KCs, such as IL-6 and Tumor necrosis factor-alpha (TNF-α), synergize with HSC-mediated ECM deposition to construct an immunosuppressive microenvironment, shielding metastatic foci from immune attack [75,76].

Beyond their individual roles, KCs and HSCs engage in a synergistic crosstalk that critically shapes the metastatic niche. This KC-HSC axis operates as a vicious cycle: Activated KCs release profibrotic and pro-inflammatory factors (e.g., TGF-β1, PDGF, IL-6, TNF-α) that are potent activators of quiescent HSCs [77]. Once activated, HSCs undergo a transformation into myofibroblast-like cells, which excessively deposit and remodel the ECM (e.g., collagen I, III), creating a stiff, fibrotic stroma that provides structural support for invading cancer cells [78]. Furthermore, activated HSCs themselves secrete a plethora of factors (e.g., CCL2, hepatocyte growth factor (HGF)) that can, in turn, reinforce the pro-tumorigenic polarization of KCs and recruit additional immunosuppressive cell*s* [79]. This reciprocal activation loop between KCs and HSCs establishes a perpetually activated, fibrotic, and immunosuppressive microenvironment that is highly conducive to the survival and outgrowth of metastatic colonies.

#### 2.2.5 Endothelial Cells and Their Angiogenesis

**Nutritional supply**

The progression of liver metastases from colorectal cancer relies on two distinct nutrient acquisition patterns: some metastatic lesions form new blood vessels through “budding angiogenesis” to supply oxygen and nutrients, which is the primary target for anti-angiogenic therapies (such as anti-VEGF antibodies) [80]. While others directly “hijack” existing hepatic vessels and attach to them for nutrient acquisition. Such metastases exhibit poor responsiveness to conventional anti-angiogenic therapies, limiting treatment efficacy [80,81].

**Metastasis**

Neovascularization plays a pivotal role in metastasis. In animal models, days 7–9 post-metastasis represent a critical window for neovascularization, directly influencing metastasis survival and expansion [32]. Sialylated immunoglobulin G (sialylated IgG) has been shown to help cancer cells move and spread in the body, including to the liver. It does this by activating a specific pathway in the cells, while anti-sialylated IgG antibodies effectively block this process [82]. Endothelial cell-associated angiogenesis is also associated with metabolic reprogramming (e.g., ketohexokinase-A (KHK-A) upregulation), further accelerating metastatic lesion growth [83].

**Immune cell recruitment**

Indeed, the process of local tumor angiogenesis does not directly attract immune cells to the tumor site. It is the concomitant microenvironmental changes that occur, such as elevated IL-10 levels and altered metabolic activity, that play a significant role, and these changes lead to a decrease in microenvironmental immune function. For example, IL-10, a key pro-metastatic factor, drives both the overexpression of PD-L1 by tumor cells and a reduction in the number of infiltrating CD8^+^ T cells and an overall weakening of the antitumor immune response, which ultimately results in liver metastases that are less responsive to immunotherapy [84].

#### 2.2.6 Lymphocytes (T Cells, B Cells, NK Cells)

**T cells**

The functional landscape of T and NK cells in CRLM paints a complex picture of local immune suppression. While the presence of specific tissue-resident memory T cells (Trm, marked by CD103^+^ and CD69^+^) is linked to better patient outcomes [85], effective CD8^+^ T cells are often scarce. Their recruitment is finely tuned by chemokine signals like C-X-C motif chemokine receptor 3 [86,87], and their function is readily suppressed by microenvironmental factors such as IL-10 [84,88]. This dysfunction is reflected in a series of aberrant checkpoint interactions: loss of activating receptors such as natural killer group 2 member D (NKG2D) [89], significant expression of PD-L1 on tumor cells [90], and inhibitory CD155-PD-1 binding [88], and it is often the continual occurrence of these changes that renders T cells dysfunctional. Therefore, reversal of this immunosuppression by interventional strategies (e.g., injection of IL-15) will allow some of the T-cell function to be restored by a mechanism that involves restoration of the expression and function of the activating receptor CD226 [88].

**NK cells**

NK cell infiltration patterns in metastatic lesions: highly cytotoxic CD49a^+^ NK cells recruit via CXCR3 and co-localize with macrophages [91], whereas liver-resident CXCR6^+^ NK cells decrease in number with downregulated T-bet expression [89] and increased granzyme K^+^ quiescent subpopulations [92]. Their functional impairment is primarily characterized by reduced effectiveness, driven by key mechanisms including decreased activity of receptors (NKG2D) and increased activity of receptors that block it [89,92]. Additionally, hepatocyte-derived fibrinogen-like protein 1 directly suppresses NK function via lymphocyte-activation gene 3 (LAG-3) [93]. Immune checkpoints such as HERV-H LTR-associating 2 and LAG-3 further exacerbate functional suppression [93,94].

**B cells**

B cells have been less extensively characterized in current studies. Still, single-cell RNA sequencing reveals a significant reduction in activated B cells within liver metastases, suggesting their potential involvement in immune regulatory defects [95].

### 2.3 Non-Cellular Components

#### 2.3.1 Extracellular Matrix (ECM)

**Components of ECM**

The ECM is mainly composed of dense matrix proteins secreted by tumor-associated cells (e.g., CAFs), of which collagen is a significant component. Its abnormal accumulation in CRLM is closely associated with drug resistance and metastasis [96]. For example, increased ECM deposition is associated with bevacizumab treatment resistance, possibly mediated through activation of fatty acid oxidation (FAO) [96]. The ECM is not only a passive scaffold but also changes dynamically during metastasis in response to evolving microenvironmental conditions. For example, TIMP1 secreted by tumor cells from EVs regulates the ECM, causing it to remodel and driving the progression of liver metastasis [97].

**Physical properties**

The physical properties of the extracellular matrix (ECM), especially stiffness, not only form a more robust structural support for tumor metastasis, but are also associated with immune escape. This stiffness stems from abnormal matrix deposition and cross-linking [98]. Atomic force microscopy (AFM) measurements have shown that ECM stiffness is significantly elevated in sorafenib-resistant patients [99]. Importantly, the assessment of tissue stiffness is no longer confined to *ex vivo* techniques. Clinical non-invasive imaging modalities, particularly magnetic resonance elastography (MRE) and ultrasound-based transient elastography (TE, e.g., FibroScan), can quantitatively measure liver stiffness in patients [100]. These techniques have been widely used to stage liver fibrosis. Emerging evidence suggests that MRE-derived stiffness parameters can also reflect the fibrotic and desmoplastic components of the TME in liver metastases, providing a potential imaging biomarker for predicting treatment response and patient prognosis [101]. Changes in the stiffness of the ECM influence the structure of tumor metastases [102,103], while enhancing metastatic potential by altering cell adhesion and pseudopod formation [103].

**Effects on the immune microenvironment**

The ECM actively steers cancer cell behavior by reshaping the physical and biochemical properties of the microenvironment. Increased matrix stiffness, for instance, provides structural support for cell adhesion while simultaneously fostering an immunosuppressive milieu that aids cancer cells in colonizing the liver and surviving there [104]. High collagen levels don’t just provide a scaffold; they directly stimulate tumor cell proliferation and enhance survival at metastatic sites, partly by protecting against anoikis—a form of cell death that usually occurs after cells lose adhesion [105].

**The impact of drug permeation**

The extracellular matrix acts as a dense physical barrier limiting drug entry. When matrix proteins such as collagen are over-deposited and cross-linked, the resulting rigidity impedes drug penetration, a phenomenon that has been well documented during bevacizumab therapy [96]. In addition to physical barriers, ECM actively activates tumor cells through pathways such as the DDR1 signaling pathway, which induces a multidrug-resistant phenotype to aid cancer cell survival [106].

#### 2.3.2 Soluble Factors (Cytokines, Chemokines, and Growth Factors)

Soluble factors usually play a mediating role. For example, transforming growth factor-β (TGF-β), which induces EMT through activation of mothers against decapentaplegic homolog (SMAD) signaling pathways (e.g., SMAD2/SMAD4), enhances the invasiveness of the primary tumor cells [107], which in turn mediates the passage of primary tumor cells across the basement membrane and thus reaches the distal organs and invades the liver (e.g., [108,109]). It induces TGF-β1 proteins, which usually further promote metastatic formation and angiogenesis after colonization of tumor cells [110]. Here are many examples of the same, such as IL-6 promoting extracellular matrix remodeling by upregulating tissue inhibitor of metalloproteinases 1 (TIMP1) expression through activation of the STAT3 pathway [111].

#### 2.3.3 Extracellular Vesicles (EVs)

**Intercellular communication**

Colorectal cancer metastasis to the liver, which is highly dependent on intercellular communication, alters the immune microenvironment of the distant organ before metastasis occurs, with EVs, especially exosomes, acting as key messengers [112]. These nanoscale carriers transport a diverse cargo of bioactive molecules, including miRNAs, cyclic RNAs, and proteins, thereby facilitating an ongoing dialog between cancer cells, local mesenchymal stromal cells, and the broader liver environment [113–115]. This exchange ultimately boosts the cancer’s invasiveness and its ability to adapt to and thrive in the distant liver tissue [114,116]. A clear example of this is seen when exosomes originating from the primary colorectal tumor travel to the liver. Upon arrival, they activate hepatic stellate cells residing in the liver and deliver specific molecules (e.g., cyclic RNA), which are the “seeds” that reconfigure the local microenvironment, thus effectively creating a suitable “pre-metastatic niche” for the invading cancer cells. The “pre-metastatic niche” is effectively developed for the invading cancer cells [112,117,118].

**Pre-metastatic niche formation**

PMN formation is the basis for subsequent immunosuppression and angiogenesis. Specific exosome components, such as HSPC111, can upregulate liver stellate cells, inducing immunosuppression and stromal remodeling to promote liver metastasis directly [119]. Furthermore, exosomes enhance the liver’s “readiness” for cancer cells by modulating the phenotypes of immune cells (e.g., macrophages and neutrophils) and promoting vascular alterations [116,119].

**Drug resistance**

Regarding drug resistance, exosomes enhance tumor survival and metastatic propensity by mediating interactions between tumor cells and stromal cells (e.g., fibroblasts and immune cells), a process closely linked to drug-resistant metastasis [120].

## 3 Pivotal Roles of TME in CRLM Progression

### 3.1 Formation of the Pre-Metastatic Niche (PMN)

The pre-metastatic niche (PMN) is a concept describing the physiological and molecular alterations in a distant organ beforethe arrival of tumor cells, which create a permissive microenvironment conducive to metastasis [121]. In the context of CRLM, the formation of the liver PMN can be summarized as a sequential process: (1) Primary colorectal cancer cells secrete factors e.g., VEGF, TGF−β and EVs into the circulation [122]. (2) These factors educate resident liver cells, including KCs and HSCs, leading to immunosuppression through the recruitment of regulatory T cells (Tregs) and MDSCs, as well as ECM remodeling [123]. (3) These changes collectively establish an immune-tolerant and structurally supportive ‘soil’ that attracts and supports the ‘seed’—circulating tumor cells—upon their arrival in the liver [124].

In fact, before metastatic cells can successfully colonize the liver, they must first establish a PMN that supports their survival. Tumor-derived exosomes appear to be key players here; their miRNAs can travel to the liver and, through cell-to-cell signals, trigger local immunosuppression and fibrosis, essentially condition the microenvironment for incoming cancer cells [116]. Once this process starts, resident liver cells, such as sinusoidal endothelial cells and stellate cells, get involved. They release pro-inflammatory factors such as VEGF and TGF-β, cytokines that remodel the ECM. This activity creates the necessary adhesion sites and growth signals that metastatic cells need to gain a foothold [125,126]. There’s also evidence that the primary tumor can suppress the liver’s immune defenses from a distance, possibly by altering local metabolic processes like tyrosine metabolism to produce immunosuppressive molecules such as kynurenine [127].

### 3.2 Cancer Cell Colonization and Growth

As described in Section 2.2.2, CAFs secrete various ECM components and growth factors, which collectively create a supportive niche for colonizing cancer cells. They actively help metastatic cancer cells survive and grow by pumping out growth factors like fibroblast growth factor 19 (FGF-19) and hepatocyte growth factor (HGF), while also building a supportive scaffold with ECM components such as collagen and fibronectin [125,128]. Single-cell RNA sequencing reveals significant heterogeneity among CAFs in liver metastases [28]. Furthermore, the liver’s unique immune-tolerant microenvironment—such as myeloid cells highly expressing PD-L1—further suppresses T cell activity, aiding cancer cells in evading immune clearance [129]. Hypoxia-inducible factor HIF-1 α is upregulated in metastatic foci, adapting to hypoxic conditions and promoting clonal expansion by activating glycolysis and angiogenesis-related genes [130,131].

### 3.3 Immune Escape

The tumor microenvironment in CRLM is effective at creating an “immune-privileged” zone that shields cancer cells from attack. One significant way this happens is through a buildup of immunosuppressive cells—TAMs, MDSCs, and Tregs flood the area, releasing molecules like IL-10 and TGF-β that directly cripple the function of cancer-fighting T cells [132,133]. Immune checkpoints compound the problem. We observe high levels of PD-L1/PD-1 and cytotoxic T-lymphocyte associated protein 4 (CTLA-4) in this space; notably, tumor-derived exosomes can even transport PD-L1 to dendritic cells, thereby contributing to T cell exhaustion [134,135]. It is also a brutal contest for resources. Tumor cells outcompete T cells for essential nutrients like glucose and amino acids; for instance, metabolizing tryptophan into kynurenine creates a local environment that directly suppresses CD8^+^ T cell activity [127,136]. Additionally, liver-specific KCs can induce immune tolerance by phagocytosing tumor antigens [137].

### 3.4 Angiogenesis and Nutrient Supply

The VEGF signaling pathway stands out as a central driver of angiogenesis within the tumor microenvironment [130]. When oxygen levels drop, HIF-1 α kicks into gear, boosting VEGF expression to lure endothelial cells and form new blood vessels, all in an effort to feed the growing metastatic site [138]. But this newly formed vascular network is often a mess. Its abnormality not only delivers nutrients but also increases permeability through molecules like ANGPT2, thereby making it easier for cancer cells to escape into the tissue [130]. Wrapping around this chaotic system, CAFs and a remodeled, fibrotic extracellular matrix create a physical shield. This barrier protects the metastatic cells from the force of blood flow and insulates them from attacks by immune cells [125,139].

### 3.5 Mediation of Drug Resistance

The push for combination therapies—aimed at crippling the tumor microenvironment by simultaneously targeting immune suppression and angiogenesis—represents a clear path forward [140,141]. Yet, the TME is notoriously adept at mounting defenses. Its dense, collagen-packed matrix physically blocks drugs from getting through, and cancer-associated fibroblasts make things worse by turning up the interstitial pressure, effectively sealing the area off [139]. But the resistance isn’t just physical. Cellular players such as TAMs and MDSCs actively protect tumors by releasing cytokines, such as IL-6. These cytokines trigger the STAT3 pro-survival pathway in cancer cells, helping them withstand treatment [132,142]. Tumors also defend against attack by remodeling metabolic mechanisms. We note that accelerated fatty acid oxidative metabolism contributes to cancer cell resistance to chemotherapy and simultaneously impairs the antitumor activity of CD8^+^ T cells. These metabolic alterations are also significant changes in tumor progression [136].

### 3.6 Resistance Mechanisms to TME-Targeted Therapies

Despite the initial promise, resistance to TME-targeted therapies remains a major clinical hurdle. The mechanisms are multifaceted and often involve the remarkable adaptability of the TME. (1) Cellular Plasticity and Compensation: Targeting one immunosuppressive population (e.g., M2-TAMs) may lead to the expansion of another (e.g., MDSCs or Tregs) to maintain an immunosuppressive milieu. Similarly, depleting specific CAF subpopulations can induce phenotypic switching in remaining CAFs, perpetuating tumor support functions [30]. (2) Metabolic Reprogramming: Tumor and stromal cells can alter their metabolic pathways to evade therapy. For instance, inhibition of angiogenesis can exacerbate hypoxia, selecting for cancer cells with enhanced glycolytic metabolism or inducing autophagy as a survival mechanism [143]. (3) ECM-Mediated Trapping and Barrier Function: The dense, cross-linked ECM not only limits drug penetration but can also sequester therapeutic antibodies, preventing them from reaching their targets [144]. (4) Evolution of Immune Evasion Mechanisms: Under the selective pressure of immunotherapy, tumor cells can upregulate alternative immune checkpoints (e.g., upregulation of LAG-3 or TIM-3 upon PD-1/PD-L1 blockade) or lose antigen presentation machinery [145]. Understanding these resistance mechanisms is paramount for designing effective combination therapies that preemptively target alternative escape pathways [143].

## 4 TME-Targeting Therapeutic Strategies: Opportunities and Challenges

### 4.1 Targeting TME Cell Components

**Figure 3:**
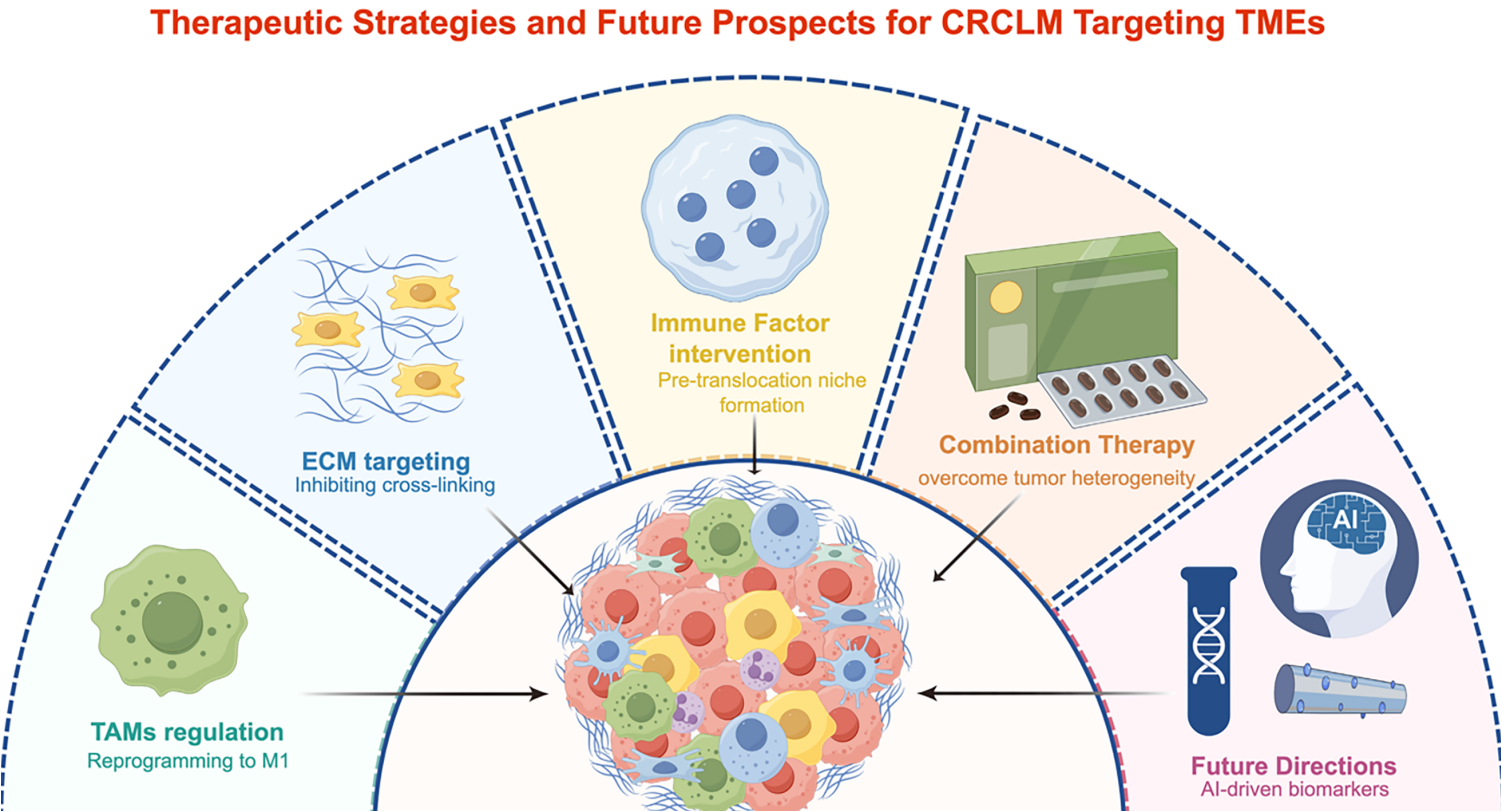
Therapeutic strategies and future prospects for CRLM targeting TMEs. Some of the therapeutic strategies currently employed are shown here, which can be directed against different TME targets, such as altering the polarization of TAMs, inhibiting cross-linking of ECM, and inhibiting the pre-metastatic state. Meanwhile, future studies can be conducted with the help of artificial intelligence and big data. CRLM, Colorectal cancer liver metastasis; ECM, Extracellular matrix; TAMs, Tumor-associated macrophages; TMEs, Tumor microenvironments.

To provide a comprehensive overview, we have summarized the diverse therapeutic landscape of CRLM in Fig. 3, which highlights how current TME-targeting strategies and emerging computational advancements are collectively shaping future clinical prospects.

#### 4.1.1 Targeting TAMs

As mentioned earlier, macrophages play a crucial role in promoting neovascularization and immunosuppression, with macrophage polarization being a key component. A recent study focused on reprogramming these cells, specifically converting tumor-promoting M2-type TAMs into cancer-fighting M1-type TAMs. The study demonstrated that targeting the **t**ripartite motif-containing 26 (TRIM26) gene can effectively alter the polarization status of tumor-associated macrophages, thereby inhibiting metastatic spread. This approach may offer a promising strategy for initiating macrophage therapy, as opposed to traditional targeted therapies [146]. The real complexity, however, lies in the diversity of these macrophages. Not only do they change location and behavior over time and space within metastatic foci, but they also contribute to cancer progression through multiple redundant mechanisms, such as the secretion of immunosuppressive extracellular vesicles. Several agents targeting TAMs, such as CSF1R inhibitors (e.g., pexidartinib (PLX3397)) and CD40 agonists, are under clinical investigation to modulate the immunosuppressive TME in various cancers, though their efficacy in CRLM specifically requires further validation [147]. This high degree of heterogeneity underscores the need to develop more precisely targeted strategies [148–150].

#### 4.1.2 Targeting CAFs

In the previous Mechanisms section, we described that CAFs promote the metastatic process by secreting ECM components such as hyaluronic acid (HA), collagen, and cytokines. Therefore, inhibiting the activation of CAFs, such as through the targeted inhibition of the HAS2 enzyme, may provide a viable strategy to suppress the formation of a fibrotic microenvironment [151]. However, the “cross-talk” between CAFs and tumor cells shows bidirectional regulation (e.g., activation of hepatic stellate cells), and such targeting is often ineffective. The impact of targeting CAFs on the repair function of normal tissues needs to be explored in depth in subsequent studies [152].

#### 4.1.3 Targeted Angiogenesis

The ECM barrier in the TME of liver metastases impedes drug penetration, thus limiting the application of anti-angiogenic drugs in CRLM [153]. The development of novel materials for drug delivery is imperative [154]. The efficacy of anti-angiogenic therapy may also be influenced by the histopathological growth pattern of the metastases, with desmoplastic HGPs potentially showing different vascular dependency compared to replacement HGPs [25].

### 4.2 Targeting Non-Cellular Components of the Tumor Microenvironment

#### 4.2.1 Redefining ECM

Since ECM remodeling may disrupt standard tissue architecture, and the dynamic changes in ECM components increase targeting difficulty [152,153], specific enzyme preparations (such as hyaluronidase) that degrade HA can still reduce CAF infiltration and metastatic activity. For example, in a fatty liver model, inhibiting HA synthesis significantly reduced metastasis [151].

#### 4.2.2 Regulating Soluble Factors

Blocking key factors (such as TGF-β and CCL2) can reverse immunosuppression [116,130]. For example, exosome microRNA-1246 secreted by CRC plays a crucial role in inducing HSC activation and reprogramming the TME. Blocking such vesicles may represent a direction for future development [116]. However, it is also necessary to consider that factor networks possess redundancy and compensatory mechanisms; single-factor blockade may yield limited therapeutic effects, necessitating more multidimensional inhibition [115].

#### 4.2.3 Targeted EVs

There is no doubt that tumor-derived EVs play a crucial role in constructing the pre-metastatic microenvironment. Consequently, research has naturally focused on blocking the release of these EVs or intercepting their signaling. Targeting the specific lncRNAs they carry, for example, has emerged as an effective way to inhibit metastasis [117,135,148]. However, a significant challenge is the diversity of EVs themselves, and we still need to clarify their different subtypes and functions more clearly to target them precisely. In addition, the inability to precisely target these tiny vesicles is also a significant challenge for the future [135]. The primary challenges include the heterogeneity of EVs, the difficulty in selectively inhibiting tumor-derived EVs without disrupting physiological intercellular communication, and the lack of efficient delivery systems for EV-targeting agents.

### 4.3 Combination Therapy Strategy

#### 4.3.1 Combination of TME Targeting with Chemotherapy/Targeted Therapy

Conventional chemotherapy often falls short because the TME is a complex barrier, characterized by low oxygen levels and an influx of immune-suppressive cells that protect the tumor [155,156]. We must contend with the TME’s physical defenses. Its dense extracellular matrix and high interstitial fluid pressure create a formidable blockade that severely limits how well any drug can penetrate the tumor core [153,154]. A more effective approach involves combining standard chemotherapeutic agents with treatments that specifically target the TME. For instance, a recent study demonstrated that pairing oxaliplatin (OXA) with a specific modulator, such as puerarin, can not only enhance its cytotoxic efficacy but also counteract its potential to foster metastasis by inhibiting chemotherapy-induced EMT [157].

#### 4.3.2 Combination of TME Targeting and Immunotherapy

The combination of targeted and immunotherapy effectively enhances immune cell function [158] while leveraging novel local drug delivery and tumor penetration capabilities to disrupt protective barriers within the TME, such as stromal fibrosis [159]. Changes that occur in the entire immune microenvironment of the metastatic tumor may explain why this combination therapy is effective. Recent studies have shown that targeting TAMs or CAFs in combination with PD-1 inhibition is effective. However, the highly tolerant immune microenvironment of CRLM, characterized by the liver’s immune-privileged nature, remains a challenge that must be overcome [135,139].

#### 4.3.3 Emerging Treatment Methods

These combination strategies provide insights into enhancing therapeutic efficacy through targeted delivery—such as modulating the liver fibrosis microenvironment—and overcoming the immune microenvironment of liver metastases may represent a significant direction for future treatments [154]. Simultaneously, targeting the TME may impair normal hepatocyte function, such as HA inhibitors disrupting matrix homeostasis [151].

#### 4.3.4 Nanodelivery Systems for TME Targeting

Nanotechnology offers a promising platform to overcome the physical and biological barriers of the TME. Smart nanocarriers (e.g., liposomes, polymeric nanoparticles) can be engineered to achieve specific targeting of TME components and controlled drug release. For instance, mannose-decorated nanoparticles have been developed to specifically deliver drugs to TAMs via mannose receptors, effectively repolarizing M2-TAMs to the tumoricidal M1 phenotype in preclinical models of CRLM [160]. Similarly, peptide-modified nanoparticles targeting fibroblast activation protein (FAP) on CAFs can co-deliver chemotherapeutic agents and CAF-inhibiting drugs (e.g., losartan), simultaneously killing cancer cells and alleviating stromal desmoplasia to enhance drug penetration [161]. Furthermore, enzyme-responsive nanoparticles that degrade upon encountering MMPs in the TME can achieve site-specific release of anti-angiogenic or immunomodulatory agents, minimizing off-target effects [162]. Although most studies are in the preclinical stage, these nanodelivery systems represent a cutting-edge translational approach to precisely modulate the TME of CRLM.

## 5 Future Directions and Outlook

**Non-Invasive Radiological Assessment of the TME**

Beyond conventional histopathological analysis, radiological imaging plays an increasingly crucial role in non-invasively characterizing the TME of CRLM, bridging the gap between basic science and clinical practice. Conventional imaging (CT, MRI) primarily provides anatomical information. However, advanced functional and quantitative imaging techniques can probe the physiological and molecular hallmarks of the TME [163]. Diffusion-Weighted Imaging (DWI): DWI measures the random motion of water molecules. The apparent diffusion coefficient (ADC) derived from DWI is inversely correlated with tumor cellularity. A low ADC value often indicates high cellular density, a feature of aggressive tumors, and has been associated with poor response to chemotherapy in CRLM [164]. Dynamic Contrast-Enhanced (DCE) MRI/DCE-CT: These techniques track the pharmacokinetics of intravenously administered contrast agents, providing quantitative parameters (e.g., Ktrans) related to tissue perfusion, vascular permeability, and angiogenesis within the TME. They are particularly relevant for monitoring the response to anti-angiogenic [165]. Radiomics and Radiogenomics: This cutting-edge field involves the high-throughput extraction of quantitative features (texture, shape, intensity) from standard medical images (CT, MRI, PET). Using radiomic analysis, these sub-visual patterns can be decoded to predict underlying TME characteristics non-invasively, such as hypoxia, immune cell infiltration (e.g., CD8^+^ T cells), fibrosis, and even specific genetic mutations (e.g., Kirsten rat sarcoma viral oncogene homolog (KRAS)) [166]. For instance, a specific radiomic signature on pre-operative CT scans has been shown to predict the immune phenotype (immune-inflamed vs. immune-excluded) of CRLM, which could potentially guide the use of immunotherapy [167]. The integration of radiological data with pathological and molecular profiles (radiogenomics) holds immense promise for creating a comprehensive, non-invasive “virtual biopsy” of the CRLM TME. This approach can enable longitudinal monitoring of TME dynamics during treatment, facilitate patient stratification, and ultimately contribute to personalized medicine.

**High-throughput single-cell sequencing and spatial omics**

Recent advances in single-cell RNA sequencing (scRNA-Seq) and spatial genomics have enabled our researchers to gain deeper insights into the tumor microenvironment in CRLM by analyzing larger datasets and incorporating more dimensions. In one clinical study, scRNA-Seq pinpointed fibroblast growth factor-19 (FGF19) as a promising therapeutic target influencing cellular crosstalk within the TME [128]. Transcriptomics at the single-cell level revealed that myofibrillar cancer-associated fibroblasts (myCAFs) drive metastasis in CRC by releasing exosomes, and helped distinguish apparent phenotypic differences between TAMs in primary colorectal tumors and their liver metastases [149]. Using spatial genomics technology, we can go one step further by classifying colorectal cancer samples into four distinct tumor microenvironmental subtypes, each defined by unique immune and mesenchymal characteristics, allowing for more precise localization of cellular subtypes [168].

**Organoids and organ-on-a-chip**

Patient-derived tumor organoids (PDTOs) retain the molecular heterogeneity of primary tumors but may lose characteristics such as consensus molecular subtypes (CMS) during culture [169]. We are seeing some of the more advanced organoid-stroma co-culture models currently available to better model complex cellular interactions in the tumor microenvironment. Researchers have already created a biobank of 50 organoids derived from CRLM, each accompanied by multi-omics data like genomic and transcriptomic profiles. This resource is proving valuable for both drug screening and improving prognostic predictions [170]. The field is moving toward even greater complexity, with newer hepatic organoids that incorporate vascular and stromal components. These advanced systems are beginning to reproduce the immunosuppressive features of the actual TME, providing a more realistic platform for testing therapies [171,172].

**Artificial intelligence and big data**

The strength of AI lies in the speed and breadth of data discovery. For example, deep learning models applied to digitized hematoxylin and eosin (H&E)-stained whole-slide images of CRC liver metastases have successfully predicted TME subtypes (e.g., immune-enriched, stromal-rich) and patient prognosis, achieving an accuracy comparable to that of molecular profiling [173]. Natural language processing (NLP) algorithms can mine electronic health records to identify clinical factors associated with specific TME features and immunotherapy response. Moreover, graph neural networks (GNNs) are being employed to model the complex “crosstalk” between CAFs and tumor cells by integrating single-cell RNA sequencing and spatial transcriptomics data, thereby identifying novel ligand-receptor interactions that could be therapeutically targeted [174]. This kind of analysis is particularly good at untangling key interaction pathways, such as the complex “crosstalk” between CAFs and tumor cells [152]. One study discovered that sialylated IgG is a potential player in metastasis, suggesting a likely new target for treating colorectal cancer liver metastases [82].

**Novel drug delivery systems and personalized and precision medicine**

On the therapeutic front, researchers are designing new delivery systems to overcome the TME’s challenges. Knowing that the environment is immunosuppressive—often polarizing Kupffer cells into an M2 state—there’s a push to develop delivery systems that specifically target macrophages to improve drug efficacy [67]. Personalization is also advancing. Biobanks of patient-derived CRLM organoids now allow for high-throughput drug screening, which can identify new uses for existing medications [175]. This move toward precision medicine means that identifying a patient’s specific TME subtype (like the CCC subtype) could directly guide whether immunotherapy or a targeted therapy is chosen [168]. Finally, a deeper understanding of specific cell types—particularly the spatial distribution of TAMs within the metastatic niche—is providing a solid rationale for designing more effective combination therapies [132].

**Accelerating translational research**

PDTO models are already proving useful in preclinical drug testing, as seen with the evaluation of the casein kinase 1 inhibitor SR30297 [176]. Their broader application, however, faces some practical hurdles—like the tendency for these organoids to lose key molecular subtypes, such as the consensus molecular subtype (CMS), after being in culture for a while. A promising path forward involves quickly merging single-cell data with organoid models. Efforts like the multi-omics analysis of CRLM already point the way [170]. This kind of integrated approach could significantly shorten the long journey from discovering a mechanism to launching a clinical trial.

**Limitations**

While this review offers a comprehensive synthesis of the TME’s role in CRLM, we must acknowledge certain inherent limitations that define the current landscape. A significant portion of our mechanistic understanding still relies on preclinical models, which may not fully capture the profound spatial and evolutionary heterogeneity seen in human patients. Looking ahead, the integration of spatial multi-omics and patient-derived organoid systems will be essential to decode the site-specific cues of the hepatic niche at unprecedented resolution. Ultimately, we envision that shifting from ‘one-size-fits-all’ approaches toward TME-stratified precision strategies will be the key to bypassing current therapeutic barriers and fundamentally transforming the clinical prognosis for patients with CRLM.

## 6 Conclusion

To this day, liver metastasis from colorectal cancer remains a challenge that must be overcome. Looking ahead, advancing research on TME heterogeneity and dynamic evolution requires leveraging single-cell/spatiotemporal multi-omics and organoid models. Ultimately, breakthroughs in CRLM treatment will be achieved through interdisciplinary collaboration, targeting precise TME modulation via combined therapeutic strategies, nanodelivery systems, and AI-assisted personalized therapies. We predict that in the future of CRLM treatment, there will be a shift from the current single, unchanging targeted therapy to a more diverse and dynamic treatment based on changes in TME composition. Of course, this will require liquid biopsy technology that monitors TME changes in real time, combined with AI algorithms to guide the sequential or combined application of these targeted agents.

## Data Availability

Not applicable.
